# Medicine for a Changing Planet: Online Clinical Cases in Planetary Health and Infectious Diseases

**DOI:** 10.1093/ofid/ofae691

**Published:** 2025-10-10

**Authors:** Seth D Judson, Kelsey Ripp, Noelle A Benzekri, A Desiree LaBeaud, Erika Veidis, Michael Xie, Alice Tin, Michele Barry, Peter Rabinowitz

**Affiliations:** Division of Infectious Diseases, Department of Medicine, Johns Hopkins University School of Medicine, Baltimore, Maryland, USA; Center for One Health, University of Global Health Equity, Butaro, Rwanda; Division of Allergy and Infectious Diseases, Department of Medicine; Department of Health Systems and Population Health, University of Washington, Seattle, Washington, USA; Division of Infectious Diseases, Department of Pediatrics, Stanford School of Medicine, Stanford, California, USA; Center for Innovation in Global Health, Stanford School of Medicine, Stanford, California, USA; Baylor College of Medicine, Houston, Texas, USA; Swedish Cherry Hill Family Medicine Residency, Department of Family Medicine, Seattle, Washington, USA; Center for Innovation in Global Health, Stanford School of Medicine, Stanford, California, USA; Departments of Environmental and Occupational Health Sciences, Global Health, University of Washington, Seattle, Washington, USA

**Keywords:** climate change, infectious diseases, medical education, One Health, planetary health

## Abstract

Planetary health and One Health have become key concepts in public health, scientific, and veterinary training, yet these concepts remain incompletely integrated into clinical medical education. Recognizing this gap, we created a free online curriculum called “Medicine for a Changing Planet,” geared toward training the next generation of health professionals in clinical skills to detect and manage the impacts of global and local environmental change on their patients. Using a case conference-style format familiar to medical trainees, we created 11 clinical case studies teaching both planetary health concepts and clinical competencies, leveraging Bloom's Taxonomy. Here, we describe the creation of these clinical cases, highlighting those centered on emerging infectious diseases that teach clinical skills related to the human–animal interface, globalization, and climate change. The online cases have been visited by more than 4000 unique users from 99 countries, piloted with residency and medical school programs, and made available for CME credit.

## Teaser

Planetary health emphasizes the impact of environmental change on infectious diseases, yet integration of this concept into clinical medical education remains limited. We describe developing online case studies to teach competencies in planetary health, including multiple cases on infectious diseases.

Climate change, environmental degradation, and multiple infectious disease epidemics threaten the health of humans, animals, and the environments that we share [1]. Although “One Health” and “planetary health” are relatively new terms to describe the relationships between human, animal, and environmental health, clinicians have recognized this connection for centuries [2]. Yet despite this recognition and the increasing need for clinicians to understand these relationships in the Anthropocene [3], formal integration of these concepts remains limited in medical education. For example, One Health is more commonly taught in veterinary schools than in medical schools [4, 5], and reviews of medical school curricula found planetary health teaching to be lacking [6–8].

Although there are ongoing efforts to increase planetary health teaching in medical schools, including multiple student-driven initiatives [6, 9, 10], gaps remain in teaching these concepts in the clinical phase of training. Given the constraints on medical curricula and increasing emphasis on clinical competencies [11], there is a need to demonstrate how concepts such as planetary health translate into clinical management of patients. Therefore, we created an online curriculum, “Medicine for a Changing Planet,” consisting of clinical scenarios based on real cases that teach both concepts and clinical competencies related to planetary health. Here, we describe the cases focusing on emerging infectious diseases and lessons learned from their development and dissemination.

## Development and Dissemination of Planetary Health Case Studies

After multiple discussions among a group of medical trainees and faculty, we recognized the need for teaching concepts and clinical competencies in planetary health. We used the Planetary Health Report Card [6] to identify potential topics. For each topic, we identified (1) planetary health learning objectives and (2) corresponding clinical competencies, using a revised version of Bloom's Taxonomy [12]. We identified 11 topics, with each topic consisting of multiple planetary health objectives and clinical competencies.

For each topic, we identified examples of real-world clinical scenarios. We chose to present the scenarios as case studies, given the familiarity and flexibility of case-based learning for medical trainees. Diversity, equity, and inclusion were key principles for designing the cases. We aimed for equal representation of cases from the Global South and for the cases to address health equity. We drafted the cases in a case conference format, similar to that frequently used by ACGME residency programs, and mapped them to corresponding ACGME milestones to enable integration into existing didactics, such as morning report or noon conference.

In addition to the traditional components of a case conference, we added additional sections to our case studies to reflect planetary health objectives and competencies. For each case we emphasized the importance of a “Social–Environmental” history, which expands on the social determinants of health to include consideration of environmental factors in history taking, as well as a “host–environment” approach to prevention. Additionally, a “Beyond the Clinic” component discusses notifying and collaborating with public health authorities, and a “Call to Action” section provides concrete steps that clinicians can take to address planetary health issues at the level of the clinic, community, and society.

We obtained feedback on the draft cases from content experts and an advisory committee and then piloted the cases with residents and medical students. We shared the revised cases via a website for greater dissemination (available at: https://www.medicineforachangingplanet.org/). Free CME credit for the cases was provided by the Stanford School of Medicine Continuing Medical Education program. In addition to the online self-paced cases, we created downloadable slide decks and self-evaluation questions that instructors can use for didactics. According to our website data, there have been more than 4000 unique users from 99 countries in 10 months.

## Infectious Disease Case Studies

Six of our cases relate to planetary health and infectious diseases. Three focus on emerging infectious diseases, covering topics on zoonoses, pandemic preparedness, and vector-borne diseases (Table 1). Additional cases describe the infectious disease risks to consider when providing care to refugees and migrants, recognizing sentinel cases in animals, and responding to water-related disasters (Table 2).

**Table 1.** Planetary Health Emerging Infectious Disease Cases

Topic	Location	Planetary Health Objectives	Clinical Competencies
Emerging zoonoses	India	Understand how ecological changes and human–animal interactions contribute to emerging zoonoses Apply knowledge of the local human–animal interface to identify and prevent emerging zoonotic diseases	Integrate history of animal contacts and environmental exposures in the development of a differential diagnosis that includes zoonoses Select appropriate diagnostic testing for emerging zoonotic diseases Understand the origins of zoonotic outbreaks including modes of transmission and sources of spillover Identify community health interventions that can limit zoonotic disease spread
Pandemic preparedness	Uganda/USA	Understand how globalization, travel, and changes to human–animal interface create opportunities for pandemics Identify sentinel cases of diseases with pandemic potential to safeguard human, animal, and planetary health	Know how to perform a comprehensive history, examination, and diagnostic testing for fever in a returning traveler Understand how to diagnose emerging zoonoses and high-consequence pathogens Apply measures to ensure healthcare and laboratory worker safety Communicate and coordinate with public health outbreak response
Vector-borne diseases	USA	Understand how climate change can affect risks for vector-borne diseases Identify sentinel cases of vector-borne disease related to climate change Understand the role of the clinician in the diagnosis, management, and prevention of vector-borne disease	Integrate history of vector exposure to develop a differential diagnosis for vector-borne disease Understand and prioritize diagnostic testing for vector-borne disease Understand populations vulnerable to vector-borne disease, integrating weather data, extreme events, and local environmental factors Access and integrate risk maps, surveillance data, and local outbreak and entomology data to improve diagnostic accuracy Educate patients, communities, and policymakers about prevention and the links between vector-borne disease and climate change

**Table 2.** Additional Planetary Health Cases Related to Infectious Diseases

Topic	Location	Planetary Health Objectives	Clinical Competencies
Pets and other animal sentinels	USA	Understand 3 reasons why animals can be “sentinels” for environmental health hazards to humans, including infectious and noninfectious hazards related to environmental change Explain 4 reasons for healthcare providers to communicate with veterinarians about health events Explain 5 ways to communicate with veterinarians about animal health and sentinel disease events related to shared environmental risks	Understand what to do when patients give history of sick animals in the house or nearby Develop communication strategies with veterinarians and animal health experts Recognize sentinel cases (human and animal) Understand how to notify regarding sentinel cases as a first step to improving shared environments
Displacement and refugee health	Somalia/USA	Define forcibly displaced persons Illustrate global migration patterns of forcibly displaced persons Recognize how environmental and political factors contribute to forced displacement and impact the health of migratory populations	Identify the leading health conditions among resettled refugees and migrant populations Access and apply the Centers for Disease Control and Prevention guidelines for immigrant and refugee health in the clinical management of a newly arrived refugee Screen for, diagnose, and treat specific health conditions of concern among refugees presenting for care Provide preventive care for resettled persons Provide culturally competent care Integrate up-to-date resources and guidelines into the clinical management of refugees and migrant patients
Water-related disasters	Philippines	Describe the link between climate change and disasters, including water-related disasters List common direct and indirect health effects due to flood and drought disasters, including both surgical and nonsurgical syndromes Understand the concept of sentinel cases (human and animal) of water-related disease Recognize role of health care providers in response to water-related disaster Understand how climate change and extreme weather events interact with healthcare/surgical systems	Take a medical history that screens for water-related health risks Screen for water-related health issues in the physical exam, including those requiring surgical intervention Detect and manage sentinel cases of water-related disease Recognize and manage necrotizing soft-tissue infections as a surgical emergency

### Emerging Zoonoses

Understanding the local human–animal–environmental interface and ecological changes are critical to diagnosing and managing zoonotic diseases. To illustrate these principles, we used clinical data from the 2018 Nipah virus outbreak in Kerala, India [13], and discussed the Bangladesh outbreaks traced to consumption of date palm sap by Pteropus fruit bats [14]. Detecting, managing, and preventing Nipah virus infection demonstrates how astute clinicians, who are knowledgeable of the local human–animal–environmental interface, can identify index cases and prevent spread with public health collaboration.

### Pandemic Preparedness

Zoonotic viruses have caused 6 of 7 Public Health Emergencies of International Concern declared by the World Health Organization, including most recently the 2022–2023 mpox epidemic [15]. Thus pandemic preparedness requires clinical awareness of risks for spillover and transmission. We based a case study on the 2008 imported case of Marburg virus disease in Colorado [16], framing the case around the diagnosis and management of fever in a returning traveler from Uganda. In addition to emphasizing the history, physical examination, and diagnostic testing for fever in a returning traveler, our case study highlights the importance of reporting suspected viral hemorrhagic fever cases to public health officials and coordinating infection prevention and control measures. Furthermore, the case highlights how increasing globalization and human–animal contact creates opportunities for future pandemics.

### Vector-borne Diseases and Climate Change

Climate change has caused significant concern among clinicians globally, with extreme heat and water-related disasters causing health emergencies [17]. The link between climate change and infectious diseases is perhaps strongest in the setting of vector-borne diseases, particularly arboviruses. Multiple studies have shown an increased risk for *Aedes*-borne arboviruses in the setting of increasing global temperatures and changing precipitation patterns [18, 19]. We built a case around the recent fatal case of locally acquired dengue in Florida to demonstrate the need for clinicians to understand exposure risks and vector spread in the setting of climate change [20].

## Discussion and Future Directions

We learned multiple lessons while creating “Medicine for a Changing Planet” that educators may consider when integrating planetary health into medical training. We found that using real-world clinical scenarios in a case-based format was an effective way to teach planetary health competencies for clinical trainees. For example, internal medicine residents at the University of Washington, who participated in the pandemic preparedness case during a teaching conference, reported that they found the case-based format more engaging and that they want more exposure to such topics.

The prevention, diagnosis, and management of infectious diseases require concepts and clinical competencies related to planetary health. More than half of the planetary health cases we created involved infectious diseases. A unifying theme throughout all of the cases is that clinicians must be trained to detect “sentinel cases.” This is especially evident in the setting of zoonotic and vector-borne diseases that are prone to cause pandemics [15]. Early clinical suspicion, diagnosis, infection control, and coordination with public health officials are critical to preventing the next pandemic. Similarly, communicating with veterinarians and understanding the local human–animal–environmental interface can improve timely detection of infectious diseases.

Overall, despite interest among clinicians in planetary health, gaps remain in integrating these concepts into medical education. Infectious diseases provide a clear opportunity to teach planetary health principles and clinical skills. Our cases provide a launching point for further efforts and resources to train clinicians to diagnose and treat patients in the setting of global environmental change.

## Notes

***Acknowledgments.*** The authors thank additional Medicine for a Changing Planet contributors, content experts, and advisory committee members for their help and feedback on the case studies.

***Financial support*.** This work was supported by the National Institutes of Health (T32 AI007291-32 to S.D.J.) and the University of Washington Center for Leadership in Medical Education Award (to N.A.B. and P.R.). The Medicine for a Changing Planet website is supported by funding from the Stanford Center for Innovation in Global Health.
